# Cerebro-Spinal Meningitis: Brain Fever

**Published:** 1858-07

**Authors:** J. W. Craig

**Affiliations:** Churchville, N. Y.


### BUFFALO MEDICAL JOURNAL

AND

### MONTHLY REVIEW.

**VOL. 14.** JULY, 1858. **NO. 2.**

### ORIGINAL COMMUNICATIONS.

ART. I.—*Cerebro-Spinal Meningitis: Brain Fever.* By J. W. Craig, M. D., Churchville, N. Y.

Messrs. Editors,— In accordance with a promise which I made to you some time ago, I herewith transmit to you an account of an epidemic known by the name of cerebro-spinal meningitis or brain fever, which has prevailed in this place, as well as in several isolated places throughout the country, during the latter part of the winter and spring of 1857–8.

On the night of February 11th, 1857, I was called to see

N. B., aged 11 years, and learned that at 5 o'clock in the afternoon, he had eaten a hearty supper, and had appeared to be in usual health, but that on retiring at 9 o'clock he complained of feeling chilly, and soon after, had a convulsion.

When I first saw him he was comatose; face suffused; pupils dilated; and he died in fifteen minutes after I arrived.

On my return I found a messenger in waiting, requesting me to visit Mr. G., æt. 40, three miles north of the village.

When I arrived, I learned from the family that he had appeared in usual health during the day, but at 7, P. M., was seized with a chill, followed by pain in the head, for which he took a dose of common cathartic pills, which operated at 10. Soon after their operation, however, he began to sink, and died at 11, four hours after the chill.

The suddenness of the attack, the mottled appearance of the skin, with the other symptoms peculiar to this disease, convinced me of the existence of a state of thing with which I had never before met.

A *post-mortem* examination having been granted, the pathological condition was disclosed, but as to the proper treatment to be pursued, we were in as much darkness as ever.

This disease, as it occurred with us, seemed to assume two forms, viz., mild and severe, or perhaps it might be denominated, cerebro-spinal typhus, mitior and gravior.

The mild cases were ushered in with a chill more or less severe, followed by fever, pain in the head, which extended along the neck and back, and accompanied with a feeling of general uneasiness. This set of symptoms generally continued from eight to twelve hours, when an intermission occurred more or less complete: but about the same time next day, there was a return of the pain and fever, but no chill—and these last two symptoms continued to occur from one day to another, the patient able in most cases to move around the house during the intermission.

The more malignant cases were also ushered in with a chill, followed by headache, vomiting, double vision, strabismus, intolerance of light and sounds, with deafness, swollen tongue, with convulsions in children, and delirium in adults. In some cases the skin presented a mottled appearance, with spots like *taches rouges*, and in others there were petechiæ; cold extremities; feeble pulse, and ultimately death. When reäction did take place, (which had necessarily to be speedy, as these cases terminate in from four to twenty-four hours from the accession of the chill) the extremities became warm, the pulse fuller, the delirium less constant, and slight febrile action manifested itself; but in the most favorable cases of this severer form, after reäction had been fully established, the pain in the head and neck continued, the head was drawn stiffly backwards, and this rigidity extended down the whole length of the back to such a degree, that to raise the lower extremities was to raise the whole body.

The morbid sensibility to light and sound continued, as also the dilated pupil and strabismus, and in many cases, at particular hours, there were convulsions and *projectile* vomiting of green and yellow substances; in addition to the above symptoms there was great restlessness, jactitation, pain in the stomach and bowels, with anorexia, which continued for a number of days.

Perhaps the following case will serve as an illustration as well as any other, the report of which I take from my note book:

I was called to see Miss R., age 16, on Tuesday, March 24th, 1857, and learned from the family that on the Sunday previous she had attended church, and had sung and played the piano during the evening, and retired seemingly in usual health. She awoke during the night feeling cold and unable to move, but soon fell asleep again. In the morning she awoke feeling much prostrated and with a severe pain in the head; double vision followed, and she soon became restless and delirious. The family physician was called, who administered a cathartic, applied a blister to the trachea, ordered a warm pediluvium and cold applications to the head.

I found her next morning in furious delirium, with dilated pupil, double vision, spasmodic vomiting, accompanied with great restlessness, so much so that it was with difficulty she could be confined in bed. Her tongue was slightly covered with a thin white coating, and her pulse was 120 per minute.

*Treatment.* Pulv. Dov., grs. v; pulv. valer., grs. iii every three hours; continue cold appl. to head.

March 25th. Had a very restless night, but the active delirium not quite so constant. Tongue heavily covered with a white coat; has constant fever; pain in head; dilated pupils; kidneys inactive.

*Treatment.* Continue that of yesterday, alternating the powders with tinct. gelsinium, xx drops, and spts. æth. nit.

March 26th. Delirium not constant, but pain in the head and neck very severe. Head is permanently drawn back, causing excruciating pain on the slightest movement; she is quite deaf; stomach rejects everything; complains of pain in back and bowels; great intolerance of light; pulse 110.

*Treatment.* Tinct. verat., iv drops, every hour until pulse becomes less frequent, with yesterday's treatment as the stomach will bear.

March 27. Pulse 80, and irregular; patient very talkative, with occasional wandering of the mind; head still drawn back and immovable; complains of pain in neck and right arm; is very restless, has occasionally slight spasms, with a peculiar moan in breathing; had a sinking *spell*, supposed to be caused by an overdose of veratria. Her tongue is cleaning, and she had had no fever since yesterday.

*Treatment.* Sulph. quinine and pulv. Dov., aa grs. iii, to be given every three hours, with infusion of valerian and *brandy* for drinks.

March 28th. Reports herself much better; had no pain in head, and thinks the stiffness in neck not so intense as it was yesterday, although any movement caused a return of pain; determined to get up this morning, which is the only symptom of mental derangement at present. Her bowels are inactive, but there is free secretion of urine; unable to bear turning on her side on account of stiffness and tenderness of neck.

Continue treatment with generous diet.

March 29th. Reports better; slept well last night; had no pain except in lower part of abdomen; perfectly rational, and can turn her head a little; complains of a sore ear caused by pressure from lying on it; head still drawn back; intolerance of light and sounds; dilated pupils, with pulse at 80; tongue moist.

Continue treatment, with hot fomentations to head and neck.

March 30th. Reports herself able to move her head more easily than yesterday; has an occasional streak of pain in head and neck; slept well last night; bowels moved by enema; appetite a little improved.

Continue treatment.

March 31st. Reports better; turned over in bed without assistance last night, and able to raise herself up on her elbow; had some pain in head and eyes.

Continue quinine and brandy.

April 1st. Reports not quite so well; had a slight chill yesterday, and now complains of headache with acute pain in neck and shoulders; hearing very acute, so that the least noise caused severe pain; felt prostrated all of yesterday; tongue dry; countenance anxious; pulse 110.

*Treatment* expectant: infusion of valerian to be given for constant drink; quinine and brandy if necessary, and Dov. powd. if she should have fever.

April 2d. About the same as yesterday; had a slight chill this morn-ng, about the same hour as yesterday, followed by fever and slight delirium; pulse 120; bowels moved four times since yesterday. She was taken up for the first time in a number of days and had her bed made.

*Treatment.* Sol. carb. ammon. in place of brandy; quinine, grs. iii; pulv. val., grs. iii; pulv. Dov., grs. iii; powder to be given every three hours.

April 3d. Reports herself very much better; slept well last night till 3

o'clock, when she awoke with severe headache and fever; has some appetite; pulse 100; tongue inclined to be dry.

Continue treatment.

April 4th. Was quite restless all day of yesterday; pulse hurried.

Continue treatment.

April 5th. Had a very restless night after 3, A. M.; can turn in bed with great difficulty on account of pain in neck; had great intolerance of light, and complained of pain in eyeballs and soreness of eyelids.

April 6th. Was called to see her at 6, A. M., and found she had been very restless the latter part of the night, complaining of constant sharp pain in head, neck and bowels, with occasional delirium; very talkative, and in the remissions of pain, inclined to sing; pulse 120; had bowels moved by enema.

*Treatment.* Gave her five drops of veratria, also a pill of morphine and assaf. Soon after this she became quiet. Pulse fell to 80, and she got some sleep.

April 7th. Was more quiet during the day yesterday, and last night.

Continue quinine, valerian and carb. ammon. If she had pain, give $\frac{1}{4}$ grain of morphine, and if restless give pill of assaf.

Aprill 11th. Has been about the same for the last four days. Pain in the head and neck have been quieted by morphine, valerian, &c.

April 12th. Has no pain in head or neck, but very sharp pain in limbs; vomited some during the morning; hearing very acute, but no dilatation of pupils; appetite good; pulse 80.

*Treatment* expectant, except morphine to control pain.

April 15th. For the last three days has been very restless, for about eight hours in twenty-four; tongue dry and inclined to be red. I learned to-day that previous to this periodic restlessness, she had had severe spasms and spasmodic vomiting for some considerable time, *i. e.*, she had had more or less of it for two or three weeks. The vomiting is not accompanied or preceded by any nausea.

*Treatment.* Morphine and valerian, with quinine and brandy, if necessary; strychnine, gr. $\frac{1}{20}$; pulv. valerian, grs. ii, to be given every six hours.

April 23d. Patient has gradually been gaining strength since the 15th, and is now able to sit up nearly an hour in twenty-four; has occasional slight spasms and vomiting in the morning. The restlessness continues at some part of the day, but does not observe the same regularity that it did formerly, and sometimes it is preceded by a chill. Pulse 82; tongue not quite so red.

*Treatment.* Continue nervines and morphine, and give three gr. pill of chinordine every three hours.

April 24th. Was called in haste this P. M., and learned that she had a very severe spasm, followed by vomiting this morning, and soon after complained of very hard pain in her head and neck with great intolerance of light and hearing very acute; to these were added subsultus, delirium, and all the bad symptoms of the disease. Her pulse was 50 in the minute, feeble and intermitting; breathing slow and accompanied with a peculiar moan; stomach rejecting almost every thing, vomited with spasmodic force, but not preceded by any nausea whatever; complained of tenderness in every part of the body, but principally over the stomach and bowels.

*Treatment.* Ordered mustard to epigastrium, and to sponge her body with a strong solution of ammonia, together with cold applications to head and neck; also to give her nervines, tonics, and carb. ammonia as they could be borne.

For a short time the pulse became more steady and the respiration natural.

*Treatment.* Pulv. val., grs. ii; bismuth, sub. nitre, gr. i; morphine, gr, $\frac{1}{8}$; fiat pulvis, to be given every three hours, and quinine and brandy as they may be necessary.

May 6th. Has continued to improve gradually, since the 24th of last month. Tongue looks quite natural, and appetite much improved; sat up about an hour to-day, and was moved into another room, but on going to bed became delirious; had pain in head and neck, with intermitting pulse, but was relieved in a short time by the use of morphine and brandy; has had occasionally slight spasms in the limbs, but without regularity, perhaps more apt to recur every other day; has had very little pain in head.

*Treat.* Infusion of valerian, columbo and quassia.

June 1st. Has continued to improve gradually; the vomiting, spasms, redness of the tongue, and pain in the head, have entirely disappeared, so that to-day (nine weeks from invasion) she was able to take an airing in a carriage. Perhaps it may be well to state that she was very fond of music and played the piano well, but on recovery, in attempting to play, she had forgotten almost every tune, and had but a very indistinct recollection of all that had transpired during her illness; but she soon regained her knowledge of music, and now, fourteen months from the commencement of report, she seems to enjoy good health, and her intellect is as vigorous as it ever was.

Was called to see D. W. B., aged 6, on the 30th of December, 1857, and learned from the family that on the morning of the 28th inst., he awoke feeling unwell, and did not attend school. In the afternoon he was taken with vomiting and constant pain in his head; also some cough, and stiffness of neck, although that was not a very prominent symptom. Dec. 29th, some domestic remedies were given as it was supposed to be a cold — but seemed no better.

Dec. 30th. Opium to allay pain, and quassia for and bitter stomachic to quiet vomiting.

Dec. 31st. Is much about the same as yesterday.

Continue treat. using pulv. Doveri.

Jan. 1st, 1858. Has vomited more since yesterday: pain in head very severe, also occasional pain in bowels, which would last for a moment.

*Treatment.* Sul. cinchona, grs. ii, every hour, and cold to head.

Jan. 2d. Has had no pain in head during the night, but returned at 7 this morning. Tongue coated with a white buff; no appetite; kidneys inactive; pulse 120; is at times delirious.

*Treatment.* Sul. cinchona, hyd. potass., and spirits nitre; cold to head.

Jan. 3d. Had but little pain since last visit; was rational all day, but some delirium at night; kidneys more active. Says he can see nothing; pupils dilated; neck stiff.

*Treatment.* Quinine, grs. ii each three hours, hyd. potass., and warm fomentations to head.

Jan. 5th. Seems brighter, and can see to-day; slept well last night, is a little delirious on waking; tongue heavily coated.

Continue treatment.

Jan. 6th. Has not had quite so much pain during the last twenty-four hours; has sharp, lancinating pains in head about once in ten minutes, that last but for a moment. Pulse 96; right pupil dilated; some subsultus.

*Treatment.* Quinine, grs. ii; valerian pulv., gr. ii, one powder to be given every three hours: with liniment of arnica. aconite, hyosciam tinct. and camphor, to be rubbed on scalp.

Jan. 7th. Had but very little pain since last visit; had a paroxysm at 7, A. M., but comfortable during the day, at 5, P. M., became semi-comatose, and could be roused with great difficulty. At 8, P. M., had four convulsions in quick succession. Remained dull during the night, but roused quite bright this morning.

Continue treatment; also beef tea and brandy.

Jan. 8th. Had a very comfortable day yesterday, but about the same, time as the night before, had another spasm.

8 o'clock, P. M. Has been very stupid all day; complains of very great pain on being roused; pulse 120; tongue heavily coated.

Continue treatment of yesterday, and give ⅛ grain morphine to quiet pain if necessary.

Jan. 9th. Had a very comfortable night; ccmplained of very little pain. Was not much delirious. Bowels moved, the discharge having a very dark appearance; tongue heavily coated, and the coat inclined to scale off; pulse hurried.

Continue treatment, with mustard to extremities if comatose.

Jan. 10th. Tongue clean and red; seemed somewhat bright in the morning, but at 10, A. M., became comatose; pupils dilated; strabismus, &c. Roused somewhat at 5, P. M., but at 10, P. M., again became comatose, and died at 5, A. M.

In this case were observed two exacerbations of pain and fever each day, one at 7, A. M., and the other at 5, P. M., which would last each about three hours, or end about 10 o'clock.

This patient was very thin in flesh at the commencement, and illy prepared to to withstand so malignant a disease.

Mrs. G., aged 35. Had complained of pain in the head and neck for a number of days. On the 21st February, 1858, was taken with a severe chill, followed by convulsions; rigidity of the muscles of the neck and back; dilated pupils; mottled skin, and coma. Was sweated by means of hot blocks, and mustard to extremities, and as soon as sweating began she was able to swallow: 2 grains of quinine with valerian and camphor, was then given each two hours, and in the course of six hours, showed some signs of consciousness.

Feb. 22d. Is rational this morning, but cannot bear the least light or sound; has strabismus, double vision, &c.; body covered with spots like *taches rouges;* pulse 120; tongue coated, white coat; complains of great pain in head and neck; head drawn stiffly back and cannot be moved. These symptoms continued about the same till the 26th, when the double vision ceased; less pain in head, and could bear sounds and light much better.

*Treatment* has been quinine, grs. ii; camphor, grs. iii, each three hours; brandy and generous diet.

March 1st. Is able to sit up some; pain in head less, and all symptoms generally better.

Continue treatment.

Miss Page, æt. 20. Attended a party on the night of Feb. 1st; awoke on the morning of the 2d, feeling chilly and having a slight headache, but supposing it to be "sick headache," to which she had been subject, walked a mile and a-half. Ate supper at 5, but soon she was worse, and at 10, P. M.: when her physician was called she was completely comatose; skin mottled; pupils contracted, eyes crossed, &c.

A cathartic was administered, which did not operate on the morning of the 3d. A counsel was held, when croton oil, with leeches to the head and neck were administered.

Saw her at 9, P. M.; found her under the action of the cathartic, also very restless; mind wandering, unable to answer questions correctly, screaming occasionally as if in acute pain, but unable to locate it; sordes on the teeth and all the bad symptoms of the disease.

Gave her quinine, morphine and brandy, but she sank and died thirty-six hours from the attack.

Mrs. H., of Adams' Basin. Dr. B. in attendance.

Was called to see her March 21st, 1858. I learned that she had attended church during the forenoon and afternoon; ate supper at 4 o'clock, in usual health, after which she wrote a letter. Feeling chilly and some pain in her head, laid down for a few moments previous to attending evening church service; but the pain her head continued, and at 7, P. M., was observed to have a convulsion, after which she became dull and could not easily be roused. These convulsions recurred once in from five to fifteen minutes, lasting from half to one minute, and were of an oposthotonotic character. With these was suffused face; dilated pupils; head drawn stiffly back, &c.

Was sweated freely, which seemed to relieve the rigidity of the neck, but had no influence in controlling the convulsions.

*Treatment.* Quinine, grs. ii, every hour; also extract of valerian. Mustard to be applied to chest and limbs.

March 22d. At 3, A. M., or three hours after commencing the use of the quinine, the spasms were arrested but had a return of them at 6, A. M., another at 12, M.

Continue treatment.

March 23d. Had a convulsion at 12 o'clock last night. Is rational, easily roused, but inclined to sleep the most of the time; complains occasionally of pain in her head; great intolerance of light and sounds.

Continue treatment, with brandy and generous diet.

March 24th. Had a convulsion last night at 12 o'clock, but is quite comfortable to-day.

*Treatment.* Quinine, gr. i; camphor, grs. iii; every two hours, with morphine and chloroform, at 12 o'clock, if necessary.

March 25th. Had symptoms of convulsion last night, when morphia and chloroform were administered with favorable effect.

Continue treatment.

March 26th. Had eight convulsions last night, commencing three hours earlier than the night before; eyesight bad, *i. e.*, is not able to distinguish objects by looking at them; pupils dilated; is very sensitive to bright lights and hissing sounds, as whispering, &c.; has sharp pains through head and neck.

*Treatment.* Quinia and valerian, with ext. hyoscyamus to control pain.

March 27th. Was very restless last night at 12 o'clock, but had no convulsion; pain in head and neck not as bad as yesterday—can move her head a little.

Continue treatment.

March 29th. Has had no convulsion since last visit; less pain; eyesight much better, although somewhat imperfect; has appetite, and feels quite comfortable.

*Treatment,* Continue quinine in small doses, also valerian, &c., as may be indicated.

I learned from Dr. B., who had charge of the case afterwards, that she continued to steadily improve, having no bad symptoms following, except one eye was inclined for some time to look upwards; but that in the course of a few weeks regained its natural position and now, May 15, seems as well in every respect as before the attack.

Mrs. B., age 32, ate supper in usual health, but when she was clearing the dishes from the table, was taken with a slight chill, and soon, as she described herself, "a sensation as if shot by a pistol pall," seized her in the back of the ueck, followed by pain in the head; immediately her head was drawn stiffly back, and all the bad symptoms of the disease manifested themselves, and which continued for a number of days.

Mrs. P. arose in the morning feeling unwell, but was able to sit up during the forenoon. At 12, M., became sleepy, and at 4, P. M., was entirely comatose, dilated pupils, &c.

Was sweated freely, and quinine and valerian given, and in twelve hours became conscious. Recovery took place in three weeks.

N. B., age 6, was unable to see or hear, and vomited green and yellow matter from the stomach; also had from three to ten convulsions a day, for six weeks. He became very much emaciated, but eventually recovered, and now, one year from the attack, seems as well as ever. There seems to be no tendency to a return in the severe form of the disease, but in the mild cases many that were attacked last year have suffered again this spring.

One marked peculiarity of these cases is the unexpectedly sudden attack, and their terrible severity. Perhaps the patient may retire to bed in usual health, and soon after falling asleep be awakened by a chill, and the family aroused by unmistakable symptoms of convulsions: and in other cases, after eating a hearty dinner, the patient be taken with a chill, soon followed by delirium, and without any premonition whatever.

Sometimes there will be sense of uneasiness for twenty-four or forty-eight hours previous to the attack, but in the majority of the severe cases the prodronic symptoms are so slight that they are not noticed. The delirium attending this disease is peculiar, in several cases having many of the general characteristics of manu a potu.

In children there is a strong tendency to pull their hair, bite their lips and fingers, &c., so that they require constant watching to prevent them from injuring themselves. They will also start from their beds suddenly as if fearful of approaching danger, and it is with great difficulty they can be persuaded to the contrary.

I shall now proceed to give some general idea of the pathological appearances presented in those six cases which proved fatal in less than forty-eight hours from the attack. All of them on removing the calvarium, presented a turgid condition of the venous system. On removing the coverings of the brain in these cases, lymph of a yellow and greenish hue was observed in the upper sulci of that organ, and in all of them there were increased quantities of lymph, with sero-purulent fluid at the base of the brain, and extending down the whole length of the spinal cord. The choroid plexus was also very much engorged, and more or less serous effusion in the ventricles. We also found softening of the base of the brain and upper portion of the spinal cord—better marked, however, in some cases than in others.

Two cases were examined which proved fatal several months after the attack. These were both of the severe form, and convalescence was very slow; still they seemed to rally, improve in strength, and gain in flesh for several weeks, so that they were able to do considerable work. I learned from the friends that after they became able to be about the house all the time, once every week or ten days, they would be attacked with a feeling of faintness, headache and vomiting, which would continue from one to six hours, when these feelings would disappear, and the patient seem to be as well as before. A succession of these attacks would come on and go off without alarming the family very much. To one of these cases I was called in the evening. On the forenoon of the same day, the patient (a female) had drawn a child in a hand wagon for about a mile; I found her with all the formidable symptoms of the disease, viz., dilated pupils, strabismus, double vision, vomiting, headache, intermitting pulse, &c., and about once in five minutes she would seem to stop breathing, and continue so for some considerable time, during which he was perfectly conscious and rational, and at last died from seeming paralysis of the muscles of the chest.

The symptoms of the other case so nearly resemble the former that a description is unnecessary.

The *post mortem* examinations of these cases revealed a softened condition of the base of the brain and upper portion of the spinal cord, with a very copious effusion of limpid fluid in the ventricles—in the one case amounting to eight, and in the other to twelve ounces. I should have mentioned that in both of these cases there was paralysis of the scalp on back of head and of the muscles on the external portions of the back of neck, and numbness to so great an extent that a comb in dressing the hair could not be felt from the first of disease.

The diagnosis in these cases is determined by the chill, followed by sudden prostration; pain in the head and neck, with the vomiting; also the periodicity observed by the disease after it had existed for a few days.

The prognosis in most cases is favorable if seen within a few hours after invasion, though in some cases the collapse and prostration that attend the disease from its onset is such that they set at defiance all remedial measures, seeming to begin where cholera ceases.

Of 129 cases that came under my observation, 12 were fatal. Death occurred in 5 cases within thirty-six hours. In 3 it took place within one week. In 1 case it took place in the fifth week; in another in the eighth; and in 2 cases it took place after several months. The mean duration of the disease is about three weeks. The continued pain in the head, stiff neck, quick pulse, delirium and coma, are unfavorable symptoms, but occasionally cases will recover by good management under the most forbidding circumstances.

This disease has been confined for the most part to low, miasmatic regions; either in the vicinity of Black Creek and its tributaries, or in the surrounding swamps and marshy lands, although a few cases came up seemingly outside of these influences, but they were of rare occurrence, and I doubt not that if the facts were known, it would be found that they had been exposed to the same exciting causes as those that lived in the vicinity of the creek.

Its first appearance was noticed soon after the melting of a large body of snow by a south wind in 1857; and the "sudden change in the weather" was the popular assigned opinion, but in 1858 no such cause could be given, and could I give you a map of the localities where it presented itself, I think it would be proved without doubt that it had been confined, in this locality at least, to miasmatic districts. It may be further mentioned in this connection, that at that time there was a prevalence of miasmatic diseases, such as intermittents and remittents of persistence and severity before unknown and these with the marked periodicity in the mild and the latter stages of the severer cases of this disease, prove conclusively to my mind that it was of miasmatic origin.

The treatment that we have adopted has been for the most part the same as for malarial diseases; and believing it to be almost, if not entire epidemical, the general rules observed in seasons of prevailing epidemics should be adopted.

Depletion in any form is not only mischievous, but absolutely dangerous; for we have observed in every case that the administration of the mildest cathartic has been followed by an alarming prostration.

If seen during the chill every means should be used to promote speedy reäction, such as the warm bath, hot drinks, brandy, ammonia, surrounding the with hot bricks, or bottles of warm water, with sinapisms to the legs, abdomen, spine, &c. After reäction has been established, the local symptoms are to be combated by cold applications to the head and neck, which must be assiduously persevered in so that reäction may not be aggravated from their too early omissions. Should the cold applications not afford relief, warm fomentations of hops and vinegar may be substituted, which are often very grateful to the patient by quieting pain and producing sleep. The vomiting can in general be relieved by a bitter stomachic infusion such as quassia, cold, or by a pill of opium; I prefer to give the opium in pill. Should these fail, an emetic will often be succussful in quieting it And as for blisters, though they seem to be indicated, I have never seen any effects from them that were salutary, but on the contrary have often seen them productive of great irritation and restlessness. Nervous symptoms are to be relieved by the use of valerian, musk, asafœtida, &c.

But the sheet anchor in these cases I have found to be sulph. quinia, in doses of 30 to 40 grs. in twenty-four hours as the stomach will bear it.

The subintrant character of this disease in its early stages will not allow us to await an intermission; but the remedy should be given in divided doses every three or four hours; and should the case be prolonged, or of the milder type, I know of nothing more effectual than Fowler's solution in doses of from three to five drops, three times a day.

The fears that are entertained (and perhaps justly sometimes) with regard to the use of quinine, brandy, opium, &c., in diseases of the brain, should in this disease be wholly discarded, for it is on their judicious and persevering use alone that we have any dependence in this dreadful malady. And we repeat, give opium to quiet pain, quinia to stop the periodicity of the disease, with brandy and nourishing diet to sustain the sinking powers of nature.

